# Making Sure We Don’t Think About Death All Day: Some Tips From Health Science & Psychology Instructors

**DOI:** 10.1093/geroni/igaf122.1421

**Published:** 2025-12-31

**Authors:** Britteny Howell, Corrie Whitmore, Calista Kern-Lyons

**Affiliations:** University of Alaska Anchorage, Anchorage, Alaska, United States; University of Alaska Anchorage, Anchorage, Alaska, United States; University of Alaska Anchorage, Anchorage, Alaska, United States

## Abstract

Courses about death and dying can take an emotional toll on students and instructors alike. While instructors may develop resources and prepare strategies to care for their students, they often neglect their own psychological needs. Cumulative stress and collective grief can lead to faculty burnout and other adverse outcomes, suggesting a need to share resources, strategies, and information with other instructors. In this presentation, we share some self-care strategies we have found helpful to continue this important work. Personal benefits to teaching courses on death and dying include addressing a pressing workforce need for this type of education, as well as the opportunity to confront our own mortality, better understand grief and cultural views of death, and prepare ourselves and our loved ones for end-of-life situations. However, the emotional burden of this work means we also need to intentionally incorporate mindfulness, activities that bring joy, and exercise into our daily lives, nurturing our support networks, and protecting boundaries for working on the course and/or end-of-life planning so it does not overwhelm us. We also welcome ideas and suggestions from participants in this session.

